# Prophylactic clip closure for mucosal defects is associated with reduced adverse events after colorectal endoscopic submucosal dissection: a propensity-score matching analysis

**DOI:** 10.1186/s12876-022-02202-3

**Published:** 2022-03-26

**Authors:** Jun Omori, Osamu Goto, Tsugumi Habu, Yumiko Ishikawa, Kumiko Kirita, Eriko Koizumi, Hiroto Noda, Kazutoshi Higuchi, Takeshi Onda, Teppei Akimoto, Naohiko Akimoto, Norio Itokawa, Mitsuru Kaise, Katsuhiko Iwakiri

**Affiliations:** Department of Gastroenterology, Nippon Medical School, Graduate School of Medicine, 1-1-5, Sendagi, Bunkyo-ku, Tokyo, 113-8603 Japan

**Keywords:** Colorectal endoscopic submucosal dissection, Mucosal closure, Adverse events

## Abstract

**Background:**

It is unclear whether prophylactic endoscopic closure after colorectal endoscopic submucosal dissection (ESD) reduces the risk of postoperative adverse events due to variability in lesion characteristics. Therefore, we conducted a retrospective study using propensity score matching to evaluate the efficacy of prophylactic clip closure in preventing postoperative adverse events after colorectal ESD.

**Methods:**

This single-center retrospective cohort study included 219 colorectal neoplasms which were removed by ESD. The patients were allocated into the closure and non-closure groups, which were compared before and after propensity-score matching. Post-ESD adverse events including major and minor bleeding and delayed perforation were compared between the two groups.

**Results:**

In this present study, 97 and 122 lesions were allocated to the closure and non-closure groups, respectively, and propensity score matching created 61 matched pairs. The rate of adverse events was significantly lower in the closure group than in the non-closure group (8% vs. 28%, P = 0.008). Delayed perforation occurred in two patients in the non-closure group, whereas no patient in the closure group developed delayed perforation. In contrast, there were no significant differences in other postoperative events including the rate of abdominal pain; fever, white blood cell count, and C-reactive protein; and appetite loss between the two groups.

**Conclusions:**

Propensity score matching analysis demonstrated that prophylactic closure was associated with a significantly reduced rate of adverse events after colorectal ESD. When technically feasible, mucosal defect closure after colorectal ESD may result in a favorable postoperative course.

## Background

Endoscopic submucosal dissection (ESD), which has been gaining wider acceptance for the treatment of superficial colorectal neoplasms due to less invasiveness and high curability potential, is however a demanding procedure, partially due to technical difficulties and higher incidence of adverse events [1]. Specifically, the rates of delayed bleeding and delayed perforation, common major adverse events after colorectal ESD, are 0.7–3.1% [2–5] and 0.3–0.7%, respectively [2, 3]. Delayed perforation is proposed to be related to excessive coagulation in the muscularis propria. Subtle bleeding is a minor adverse event observed after ESD in some patients. Although not a clinically relevant presentation invariably requiring endoscopic intervention, hematochezia may cause prolonged hospitalization or re-admission. Therefore, prevention of postoperative bleeding should be aimed regardless of the volume of blood loss.

Mucosal defect closure using endoscopic clips after endoscopic resection is expected to reduce the rate of delayed bleeding. Several large retrospective studies showed that prophylactic clipping closure for > 2 cm lesions was beneficial for preventing delayed bleeding after polypectomy or endoscopic mucosal resection (EMR) [6, 7], whereas several randomized controlled trials demonstrated that clip placement after polypectomy or EMR did not prevent delayed bleeding [8–11]. Additionally, a meta-analysis found that the impact of prophylactic clipping after colorectal endoscopic resection for polyps < 2 cm was unexpectedly small [12], which might have been due to the lower rate of postoperative bleeding observed in small polyps. Alternatively, few reports investigated the utility of clip closure after colorectal ESD [13–16], since the closure of large mucosal defects by clipping after colorectal ESD is considered technically challenging. Several closure techniques for large mucosal defects after ESD have been recently introduced with the aim to reduce postoperative adverse events after ESD [17–22], and closeable target lesions have been expanded in size and location; however, some lesions remain difficult or impossible to completely close.

We introduced the prophylactic clip closure for post-ESD mucosal defects in practice since April 2018, expecting the preventative effect for delayed adverse events. After a transitional period, we are trying to perform it for all lesions if considered technically possible. We therefore conducted a retrospective study with propensity-score matching to evaluate the efficacy of prophylactic clip closure for the prevention of postoperative adverse events after colorectal ESD.

## Methods

### Study design and patients

In all, 242 patients with 259 superficial colorectal neoplasms underwent ESD between January 2018 and August 2020 at the study hospital. All ESD procedures were performed by five experienced endoscopists in this unit. For this study, we excluded cases of multiple lesions that were simultaneously treated by ESD (33 lesions in 16 patients) to clearly identify lesions responsible for the study outcomes; furthermore, seven non-adenomatous lesions (three neuroendocrine tumors, two mucosa-associated lymphoid tissue lymphomas, one ganglioneuroma, and one ulcerative colitis-related dysplasia) were excluded due to possible differences in vascularity. Consequently, 219 colorectal neoplasms in 219 patients were enrolled in this retrospective cohort study.

All lesions were diagnosed as node-negative cancer by white-light endoscopy followed by magnifying endoscopy with narrow-band imaging and chromoendoscopy and were classified as appropriate lesions for treatment with ESD based the Japan Gastroenterological Endoscopy Society guidelines for colorectal ESD [23]. All patients undergoing ESD were fully informed regarding the need for treatment, ESD risks and benefits, and alternative treatments including EMR and surgical resection and provided written informed consent to undergo ESD. Regarding the continuation of antithrombotic agents, we followed the "Guidelines for gastroenterological endoscopy in patients undergoing antithrombotic treatment”. of the Japan Gastroenterological Endoscopy Society; additionally, we judged to take antithrombotic agents continuously or withdrawn [24, 25]. This study was approved by the medical ethical committees of Nippon Medical School. (Approval No. 30-02-1077).

### Endoscopic submucosal dissection

All procedures were performed using a standard colonoscope (PCF-H290ZI or PCF-Q260AI; Olympus Co. Ltd, Tokyo, Japan) under insufflation with carbon dioxide. The disposable distal attachment was mounted onto the tip of the endoscope. An electrosurgical current generator (VIO300D; Erbe Elektromedizine, Tubingen, Germany) was used for electrical dissection and coagulation. The DualKnife (KD-655Q; Olympus) or the FlushKnife (DK2620J; Fujifilm Co. Ltd., Tokyo, Japan) knife was used for primary electrocauterization, and hyaluronic acid solution was used as the injection liquid. The tip of the electrocautery knife or hemostatic forceps was used for intraoperative bleeding and prophylactic hemostasis for visible nonbleeding vessels as well as for cauterization of the mucosal defect immediately after the resection. Patients with favorable physical examination, blood test, and X-ray results were allowed to drink water on postoperative day (POD) 1 and to have soft food on POD 1 or 2 following ESD. Patients with noticeable bloody stools were defined as experiencing delayed bleeding and underwent endoscopic hemostasis. On POD 3 or later, all the patients were discharged and told to monitor whether bleeding occurred during the interval between discharge and one-month outpatient follow-up visit. Emergence of abdominal pain and free air in the abdominal space in the absence of intraoperative perforation by imaging modalities was used to define delayed perforation.

### Prophylactic clipping

Since April 2018 (the initial one year as a transitional period), prophylactic clipping was performed for all lesions in which complete closure was considered to be technically achievable by the endoscopist regardless of muscular damages or defect size. Mucosal defects remained open in cases where the endoscopist determined that clip closure was difficult or should not be performed due to large size, long ESD duration, and inability to remove the lesion *en bloc.*

**Fig. 1:**
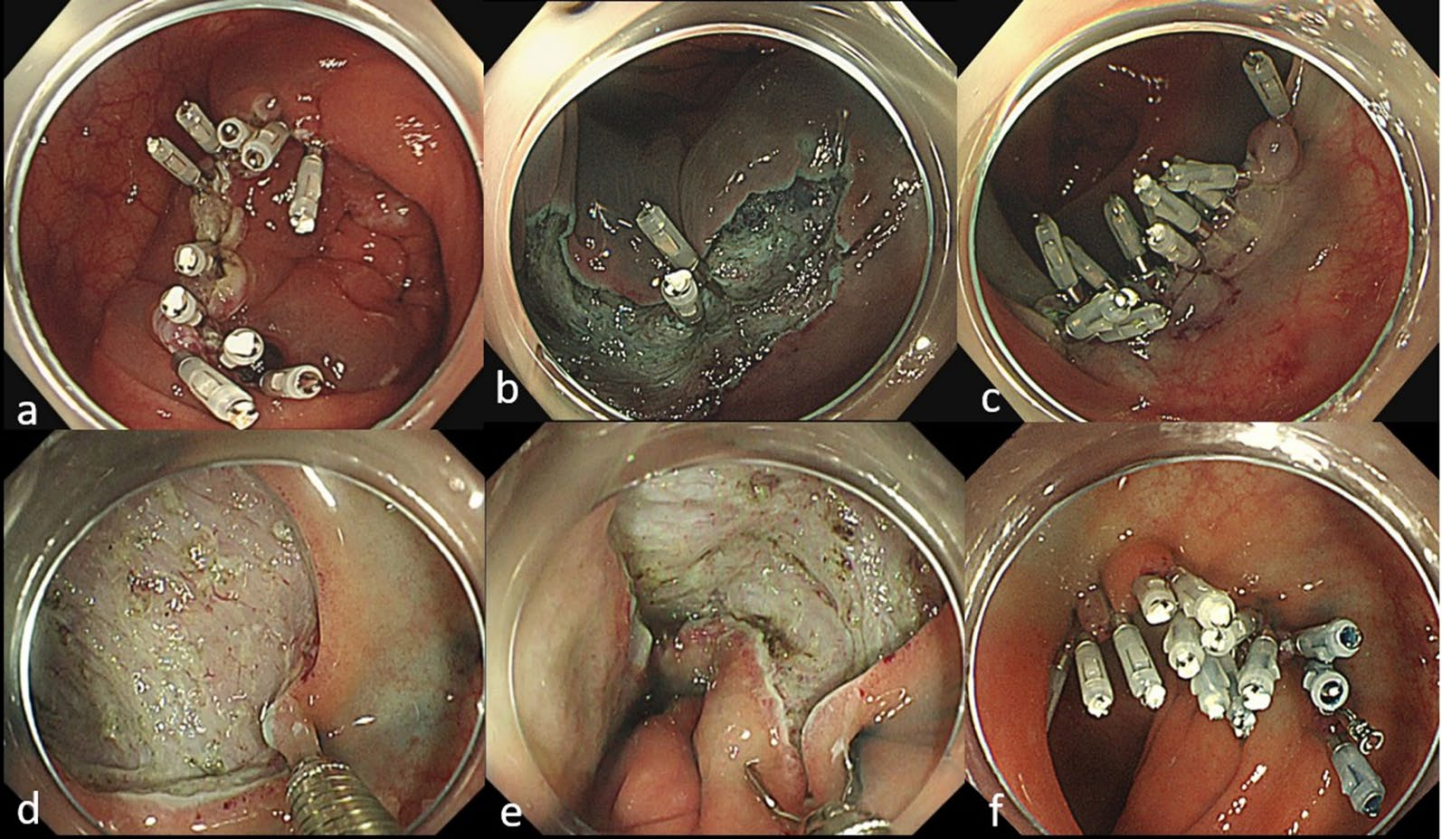
Endoscopic clip closure techniques used in the present study. **a** Conventional method. Complete closure is achieved with conventional clips by directly bridging the two mucosal rims. **b**, **c** Mucosa-submucosa clip closure method. The first clip is placed at the edge of the mucosal defect (**b**). Additional clips are placed to achieve complete closure (**c**). **d**, **e**, **f**. Hold-and-drag closure technique. The anal side of the mucosal defect is held with the repositionable clip **d**. After gently reopening the clip, both edges are grasped (**e**). The closure is completed using one repositionable clip and several standard clips (**f**)

For the closure of post-ESD mucosal defects, EZ clips (HX-610-090L; Olympus) and repositionable clips (Zeo clips; Zeon Medical Inc., Tokyo, Japan or Quick Clip Pro; Olympus) were used. First, the intraluminal air was suctioned to bring the two mucosal edges closer together. Next, one arm of the clip was attached to the proximal mucosal edge, followed by hooking of the other clip arm to the distal mucosal edge using the endoscope. Then, the clip was gently closed and confirmed to correctly grasp both edges. The closure was completed by repeating the procedure to bridge the remnant gaps between the two edges (Fig. 1a). In cases where the edges were too far from each another for successful closure using clips, half of the defect was initially closed by clips to bridge the mucosal edge and submucosal surface at the center of the defect, followed by the closure of the remaining half of the mucosal edges by clipping (Fig. 1b, c). If the clipping procedure failed, the closure of mucosal defect was abandoned, and terminated the procedure keeping the mucosal defect not completely closed.

### Outcome measures and propensity-score matching

The study patients were allocated to the closure and non-closure groups. The closure group included patients in whom the mucosal defect was completely closed by one or more clips that were placed at > 5-mm intervals. The non-closure group included patients in whom clip closure was not attempted or was incomplete due to technical difficulties.

To investigate the efficacy and safety of prophylactic clip closure for mucosal defects, postoperative bleeding and perforation after colorectal ESD were evaluated as the primary endpoint. Postoperative bleeding was categorized as major and minor bleeding. Major bleeding was defined as the presence of clinical evidence for bleeding manifesting as hematochezia requiring endoscopic hemostasis, and minor bleeding was defined as the presence of a small amount of hematochezia monitored without endoscopic intervention. Data on abdominal pain, fever defined as > 37.5℃, C-reactive protein level, and white blood cell (WBC) count on POD 1 as well as data on appetite loss, defined as < 50% meal intake on POD 2, were evaluated as secondary endpoints.

**Fig. 2:**
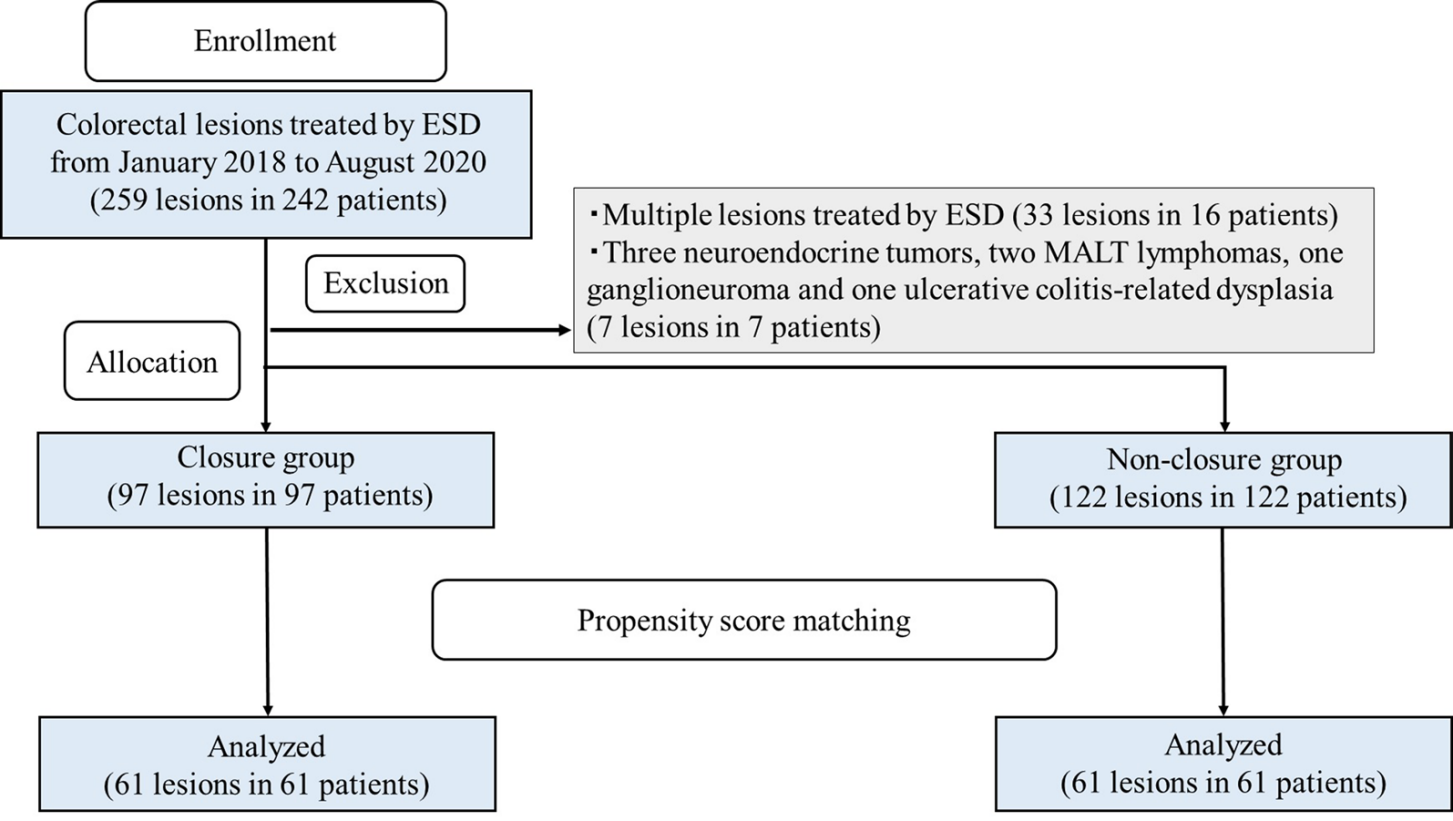
Flow diagram showing patient selection for the study. The patients were allocated into the closure and non-closure groups, which were compared after propensity-score matching. ESD, endoscopic submucosal dissection

As shown in Fig. 2, the two groups were compared after propensity-score matching to control for factors that might influence ESD treatment outcomes and adverse events. To assess these potential factors, univariate analyses were performed with the explanatory variables of age, sex, antithrombotic agents administered, lesion location, specimen size, tumor size, and procedure time of ESD. To minimize inherent bias, the two groups were matched in a 1:1 ratio, with 61 patients in each group, and propensity-score matching was used to adjust for the six covariates determined by the univariate analyses, including antithrombotic agents administered, lesion location, morphology, specimen size, tumor size, and procedure time of ESD. The caliper width of the matching was 0.0456.

### Statistical analysis

Pearson’s chi-square test or Fisher’s exact test was used to analyze categorical data for comparison of proportions. Student’s *t* test was used for unpaired data to determine differences in means between two groups. Differences were considered statistically significant with a P value of < 0.05. Data were evaluated using the SPSS statistical software package version 25 (IBM, New York, NY, USA).

## Results

### Analysis before propensity-score matching

Among the 219 lesions, 97 and 122 were allocated to the closure and non-closure groups, respectively (Fig. 2). Table 1 shows the patient characteristics before propensity score matching. The mean patient age was 69.8 ± 10.7 years in the closure group and 69.7 ± 11.3 years in the non-closure group. There were significant differences in the proportion of patients treated with antithrombotic agents (26% vs. 15%, P = 0.041), proportion of 0-IIa lesions (78% vs. 89%, P = 0.041), proportion of rectal lesions (8% vs. 31%, P = 0.001), mean specimen size (40.1 ± 12.1 vs. 47.7 ± 16.1 mm, P < 0.001), mean tumor size (26.8 ± 11.3 vs. 34.8 ± 16.6 mm, P < 0.001), and procedure time of ESD (61.0 ± 29.0 vs 85.3 ± 48.7 min, P < 0.001) between the closure and non-closure groups, respectively. The proportion of patients with concurrent lesions removed by EMR or polypectomy was not significantly different between the two groups. The *en bloc* resection in the closure and non-closure groups were 100% and 98%, respectively, with no significant difference between the two groups. In the closure group, the mean procedure time for closure was 12.4 ± 5.2 (range, 4.0–25.0) minutes and the mean number of clips required for closure was 9.8 ± 3.2 (range, 3–21).

**Table 1:** Characteristics of patients/lesions before propensity-score matching

	Closure group	Non-closure group	
	(97 lesions in 97 patients)	(122 lesions in 122 patients)	P value
Patients			
Age, mean ± SD (range), years	69.8 ± 10.7 (42–89)	69.7 ± 11.3 (39–88)	0.962
Sex, male/female	64/33	74/48	0.418
Antithrombotic agents, n (%)	25(26%)	18 (15%)	0.041
Lesions			
Location (colon/rectum)	85/12	84/38	0.001
Morphology (0–I/0–IIa)	21/76	14/108	0.041
Histology (adenoma/M/SM1/SM2 or more/other)	32/48/8/8/1	28/67/10/9/8	0.388
Specimen size, mean ± SD (range), mm	40.1 ± 12.1 (18–92)	47.7 ± 16.1 (25–101)	< 0.001
Tumor size, mean ± SD (range), mm	26.8 ± 11.3 (10–87)	34.8 ± 16.6 (10–92)	< 0.001
Simultaneous removal of other polyps*, n (%)	3 (3%)	5 (4%)	1.000
ESD outcomes			
Procedure time of ESD, mean ± SD (range), min	61.0 ± 29.0 (12–171)	85.3 ± 48.7 (11–274)	< 0.001
*En bloc* resection rate (%)	100	98	1.000
Perforation during ESD, n (%)	3 (3%)	1 (1%)	0.324
Outcomes of prophylactic clip closure			
Procedure time, mean ± SD (range), min Number of clips, mean ± SD (range)	12.4 ± 5.2 (4.0–25.0) 9.8 ± 3.2 (3–21)	– –	

SD: standard deviation

ESD: endoscopic submucosal dissection

*Endoscopic mucosal resection or polypectomy performed for concomitant lesions

The outcome data are presented in Table 2. The total number of adverse events after colorectal ESD, including delayed perforation and post-ESD bleeding, was significantly higher in the non-closure group than in the closure group (28% vs. 6%, P < 0.001). Similarly, the proportion of patients with fever was significantly higher in the non-closure group than in the closure group (12% vs. 2%, P = 0.019). However, there were no significant differences in the proportion of patients with abdominal pain, WBC count, C-reactive protein levels, and appetite loss between the two groups.

**Table 2:** Comparison of outcomes before propensity-score matching

	Closure group	Non-closure group	P value
	(N = 97)	(N = 122)	
Postoperative adverse events, n (%)	6 (6%)	35 (28%)	< 0.001
Delayed bleeding, n (%)	6 (6%)	33 (27%)	< 0.001
Minor bleeding, n (%)	5 (5%)	25 (20%)	0.001
Major bleeding, n (%)	1 (1%)	8 (7%)	0.041
Delayed perforation, n (%)	0	2 (2%)	0.504
Abdominal pain (POD 1), n (%)	11 (11%)	13 (11%)	0.872
Fever (POD 1), n (%)	2 (2%)	12 (10%)	0.019
Appetite loss (POD 2), n (%)	1 (1%)	6 (5%)	0.136
C-reactive protein (POD 1), mean ± SD (range)	0.93 ± 1.7 (0.03–11.8)	0.74 ± 1.1 (0.03–5.9)	0.316
White blood cell counts (POD 1), mean ± SD (range)	7746 ± 2182 (2200–16,400)	7716 ± 2975 (2100–29,100)	0.931

POD: postoperative day

### Propensity-score matching analysis

Propensity score matching controlled for differences in the proportion of patients taking antithrombotic agents, lesion location, morphology, specimen size, tumor size, and procedure time of ESD, which were significantly different between the two groups by univariate analysis. Consequently, propensity score matching created 61 matched pairs (Fig. 2). This model yielded a c-statistic of 0.776, indicating its ability to differentiate between the two groups.

Table 3 shows the characteristics of the two groups after propensity-score matching. The groups were similar in age (68.8 ± 11.0 vs. 68.5 ± 11.9 years, P = 0.890), proportion of males (62% vs. 62%, P = 1.000), proportion of patients taking antithrombotic agents (23% vs. 21%, P = 0.827), proportion of rectal lesions (13.1% vs. 13.1%, P = 1.000), specimen size (41.5 ± 11.9 vs. 42.9 ± 10.7 mm, P = 0.503), tumor size (29.1 ± 11.6 vs. 29.2 mm ± 12.1, P = 0.954), and procedure time of ESD (66.5 ± 28.1 vs. 68.3 ± 30.6 min, P = 0.731). Table 4 lists the outcomes after propensity score matching. Regarding adverse events, two patients in the non-closure group experienced post-ESD delayed perforation whereas no patient in the closure group developed post-ESD delayed perforation. The rate of adverse events, including delayed bleeding and delayed perforation, was significantly lower in the closure group than in the non-closure group (8% vs. 28%, P = 0.008). Furthermore, the delayed bleeding rate was significantly lower in the closure group than in the non-closure group (8% vs. 25%, P = 0.025). In contrast, there were no significant differences in the proportion of patients with abdominal pain, fever, WBC count, C-reactive protein levels, and appetite loss between the two groups.

**Table 3:** Characteristics of patients/lesions after propensity-score matching

	Closure group non-closure group		P value
	(61 lesions in 61 patients)	(61 lesions in 61 patients)	
Patients			
Age, mean ± SD (range), years	68.8 ± 11.0 (42–86)	68.5 ± 11.9 (41–84)	0.890
Sex, male/female	38/23	38/23	1.000
Antithrombotic agents, n (%)	14 (23%)	13 (21%)	0.827
Lesions			
Location (colon/rectum)	53/8	53/8	1.000
Morphology (0–I/0–IIa)	11/50	9/52	0.625
Histology (Adenoma/M/SM1/SM2 or more/other)	20/32/4/4/1	19/33/4/4/1	0.843
Specimen size, mean ± SD (range), mm	41.5 ± 11.9 (23–92)	42.9 ± 10.7 (27–80)	0.503
Tumor size, mean ± SD (range), mm	29.1 ± 11.6 (13–87)	29.2 ± 12.1 (14–68)	0.954
Simultaneous removal of other polyps*, n (%)	2 (3%)	2 (3%)	1.000
ESD outcomes			
Procedure time of ESD, mean ± SD (range), min	66.5 ± 28.1 (19–157)	68.3 ± 30.6 (11–197)	0.731
*En bloc* resection rate (%)	100	100	1.000
Perforation during ESD, n (%)	3 (5%)	0	0.244

SD: standard deviation, ESD: endoscopic submucosal dissection

*Endoscopic mucosal resection or polypectomy performed for concomitant lesions

**Table 4:** Comparison of outcomes after propensity-score matching

	Closure group non-closure group		P value
	(N = 61)	(N = 61)	
Postoperative adverse events, n (%)	5 (8%)	17 (28%)	0.008
Delayed bleeding, n (%)	5 (8%)	15 (25%)	0.025
Minor bleeding, n (%)	4 (7%)	12 (20%)	0.032
Major bleeding, n (%)	1 (2%)	3 (5%)	1.000
Delayed perforation, n (%)	0	2	0.496
Abdominal pain (POD1), n (%)	7 (12%)	6 (10%)	0.769
Fever (POD 1), n (%)	2 (3%)	7 (11%)	0.163
Appetite loss (POD2), n (%)	1 (2%)	3 (5%)	0.619
C-reactive protein (POD 1), mean ± SD (range)	0.75 ± 1.17 (0.03–7.6)	0.67 ± 1.13 (0.03–5.9)	0.724
White blood cell count (POD 1), mean ± SD (range)	7598 ± 1955 (2200–12,500)	7849 ± 3564 (2100–29,100)	0.631

POD: postoperative day

## Discussion

In the present study, we demonstrated using propensity score matching analysis that prophylactic clip closure for mucosal defects after colorectal ESD was associated with reduced postoperative adverse events including minor delayed bleeding. Although our findings cannot be generalized regarding technical difficulties, the results suggest that mucosal closure may effectively reduce postoperative adverse events and, therefore, may provide a safer and more comfortable ESD, when complete closure is achieved.

Several randomized controlled trials and one meta-analysis on prophylactic clip closure after EMR/polypectomy for small lesions showed that this approach was not beneficial in preventing delayed bleeding [8–12]. Indeed, the Japan Gastroenterological Endoscopy Society guidelines for ESD/EMR state that prophylactic clipping [23], which may have a limited effect after the endoscopic resection of colorectal polyps, may be effective in patients at high risk of postoperative bleeding, such as those with large lesions and those receiving antithrombotic therapy; however, the evidence level is low. Particularly in EMR/polypectomy for small lesions, delayed bleeding may not be sufficient to prove the efficacy of prophylactic clip closure [12]. In contrast, the risk of adverse events is considered to be higher after ESD compared with EMR. Nevertheless, to date, no study has extensively evaluated the utility of prophylactic clipping.

The lack of evidence on the utility of prophylactic clipping after colorectal ESD may be related to the fact that clip closure is technically difficult and its success depends largely on lesion characteristics. In practice, clip closure is attempted after empirical confirmation that the clip is technically durable; therefore, we acknowledge the apparent selection bias in clip-closure applications. As expected, there were significant differences in lesion characteristics between the closure and non-closure groups. Therefore, we conducted propensity score matching to analyze the true efficacy of prophylactic clip closure without the confounding effects of technical issues. The present study results suggest that defect closure after colorectal ESD should be considered in all patients whenever feasible. Furthermore, these results suggest that other closure techniques, such as endoscopic suturing, may also be effective.

The present study found that endoscopic closure using conventional clips reduced the risk of delayed perforation and delayed bleeding including minor bleeding. Major bleeding is primarily considered as a post-ESD adverse event; however, other subclinical adverse events, such as minor bleeding, that are problematic and stressful for patients may require treatment. Moreover, it can be challenging for medical staff to decide whether close monitoring by hospitalization and/or emergency endoscopy should be performed in patients with minor bleeding. However, delayed perforations are extremely severe. Nevertheless, evaluation in large cohorts is difficult, since delayed perforations are rare, and effective countermeasures remain unknown.

In the present study, minor bleeding and delayed perforation were included as postoperative adverse events, and our analyses revealed that prophylactic clip closure may prevent unexpected events including these two undoubtedly severe events. Although prophylactic clip closure required an extra cost of clips and extra time of clipping after ESD, reduction of risk after ESD by clipping is more beneficial than prophylactic clip closure since it is a less invasive treatment option. Furthermore, clip closure is a conventional technique for experienced endoscopists and clip hemostasis is superior, since muscle layer damage is prevented.

Post-polypectomy syndrome is characterized by local peritoneal inflammation in the absence of apparent perforation that can develop after colorectal ESD. This presentation is related to excessive coagulation in the muscularis propria and to large mucosal defects after colorectal ESD. Some patients have localized abdominal pain, fever, leukocytosis, and appetite loss. In the present study, there were no significant differences in the proportion of patients with abdominal pain, those with fever, WBC count, and C-reactive protein level, and appetite loss between the closure and non-closure groups, which suggested that post-polypectomy syndrome occurred equally between the two groups and that clip closure did not affect the rate of this syndrome in the present study.

Various closure devices and methods, such as slip knot clip suturing [17–19], endoscopic mucosa-submucosa clip closure [20, 21], overstitch system [26], and endoscopic hand suturing [27, 28], have been reported for the closure of artificial wounds after endoscopic resection. In the present study, we occasionally performed the endoscopic mucosa-submucosa clip closure method in ESD for mucosal defects that were too large to be closed in a single action (Fig. 1b, c). Additionally, we chose repositionable clips and performed the “hold-and-drag” closure technique in all cases [22] (Fig. 1d, e, f). Every technique has advantages and disadvantages; therefore, the selection of the closure technique depending on the situation and the endoscopist’s preference should be acceptable to achieve complete closure. Although lesions that were easily closed by clip may often be easily resected by ESD due to their small size, specific types of small lesions with severe fibrosis, abundant blood vessels, or bleeding may be difficult to resect by ESD. Therefore, clip closure may be effective for postoperative adverse events, since specific types of small lesions with severe fibrosis, abundant blood vessels, or bleeding have a high rate of muscle layer damage during ESD, even if the mucosal defects after ESD are small.

The present study has several limitations. First, the sample size was small; therefore, studies with larger cohorts are needed to confirm the efficacy of endoscopic closure. Second, we were unable to directly compare the efficacy of prophylactic clipping between the closure and non-closure groups, and prospective randomized studies are needed to establish evidence. Third, clip closure for large mucosal defects after colorectal ESD requires certain endoscopic skills and experience. Fourth, clip closure may fail for lesions in certain locations and situations. Large lesions are typically more difficult to close than small lesions. Therefore, it is increasingly difficult to evaluate the efficacy of prophylactic clips for large lesions. Furthermore, in our study, large lesions were likely to be removed by propensity score matching due to the characteristics of the dataset.

## Conclusion

Propensity score matching analysis demonstrated that prophylactic clip closure was associated with significantly reduced total adverse events after colorectal ESD. This result should be confirmed with prospective randomized trials.

## Data Availability

The datasets used and analyzed in the current study are available from the corresponding author on reasonable request.

## Abbreviations

ESD: Endoscopic submucosal dissection
MALT: Mucosa-associated lymphoid tissue
EMR: Endoscopic mucosal resection
POD: Postoperative day
WBC: White blood cell
SD: Standard deviation
