# Correction to: Prophylactic clip closure for mucosal defects is associated with reduced adverse events after colorectal endoscopic submucosal dissection: a propensity-score matching analysis

**DOI:** 10.1186/s12876-022-02259-0

**Published:** 2022-04-26

**Authors:** Jun Omori, Osamu Goto, Tsugumi Habu, Yumiko Ishikawa, Kumiko Kirita, Eriko Koizumi, Hiroto Noda, Kazutoshi Higuchi, Takeshi Onda, Teppei Akimoto, Naohiko Akimoto, Norio Itokawa, Mitsuru Kaise, Katsuhiko Iwakiri

**Affiliations:** Department of Gastroenterology, Nippon Medical School, Graduate School of Medicine, 1-1-5, Sendagi, Bunkyo-ku, Tokyo, 113-8603 Japan

## Correction to: BMC Gastroenterology (2022) 22:139 https://doi.org/10.1186/s12876-022-02202-3

After publication of this article [1], the authors reported a number of errors.

In Fig. 1, "d" should be "(d)".

In Table 1, add space in "25(26%)".

In Table 2, move "P value" one level down (as in Table 1).

In Table 2, remove spaces in "13 (11%)".

In Tables 3 and 4, remove the line below “Closure group…” (as in Table 1).

In Tables 3 and 4, move “Non-closure group” to the right (as in Table 1).

In Tables 3 and 4, move "P value" one level down (as in Table 1).

In Table 4, line “Delayed perforation, change "2" to “2 (3%)".

The original article [1] has been updated.
